# Er:YAG laser biofilm removal from zero-gap periodontal/peri-implant model system mimicking clinical attachment loss

**DOI:** 10.1117/1.JBO.30.2.025002

**Published:** 2025-02-25

**Authors:** Marko Volk, Dominik Šavli, Katja Molan, Saša Terlep, Špela Levičnik-Höfferle, Mojca Trost, Boris Gašpirc, Matjaž Lukač, Matija Jezeršek, David Stopar

**Affiliations:** a University of Ljubljana, Department of Microbiology, Biotechnical Faculty, Ljubljana, Slovenia; b University of Ljubljana, Faculty of Mechanical Engineering, Ljubljana, Slovenia; c University of Novo Mesto, Faculty of Health Sciences, Novo Mesto, Slovenia; d Fotona d.o.o., Ljubljana, Slovenia; e University of Ljubljana, Department of Oral Medicine and Periodontology, Medical Faculty, Ljubljana, Slovenia; f Institut Jozef Stefan, Ljubljana, Slovenia; g University of Ljubljana, Faculty of Mathematics and Physics, Ljubljana, Slovenia

**Keywords:** Er:YAG laser, photoacoustic removal, biofilm, attachment loss, *in vitro* model

## Abstract

**Significance:**

Here, we present a photoacoustic method to remove biofilms from periodontal and peri-implant-constrained geometries.

**Aim:**

We aim to remove biofilms from narrow periodontal and peri-implant model systems with the application of Er:YAG ultrashort laser pulses.

**Approach:**

Construction of zero-gap model system from PDMS and titanium, growth of biofilms on titanium surfaces, and removal of biofilms with Er:YAG USP, 20 mJ, 15 Hz, and 10 s were performed.

**Results:**

The results suggest that geometry, the vertical position of the laser fiber tip, and the evolution of the primary cavitation bubble significantly affect cleaning effectiveness. Cleaning was higher in the wedge part of the model system. In the zero-gap part of the model system, biofilm cleaning effectiveness was highest at the position of the laser fiber tip and decreased above and below the fiber tip. The dimension of the space in which the cavitation bubble develops determines the size and dynamics of the expanded cavitation bubble and consequently the biofilm cleaning effectiveness.

**Conclusions:**

The obtained results suggest a very good biofilm removal effectiveness in difficult-to-reach narrow geometries mimicking clinical attachment loss in the periodontal/peri-implant pocket.

## 1 Introduction

Biofilms play a pivotal role in the etiology of periodontal and peri-implant diseases.^1^ Periodontal and peri-implant pocket formation is related to an increased load of sub-gingival microorganisms on the tooth or implant surface as well as a shift in the composition of the microbial community.^2^

Plaque-induced gingivitis is an inflammatory response of the gingival tissues that is a consequence of the deposition of bacterial plaque at and below the gingival margin. The first transition from healthy to plaque-induced gingivitis may not be clinically discernible. However, clinical signs and symptoms become apparent as plaque-induced gingivitis advances to more established types of the disease. Blood in the saliva, gingival edema and redness, halitosis, and bleeding after tooth brushing are signs that patients may see in the case of established forms.^3^

Periodontitis, a chronic multifactorial inflammatory disease associated with dysbiotic plaque biofilms and characterized by the progressive loss of tooth-supporting tissues, can develop if gingivitis is left untreated. Its key characteristics include the absence of periodontal tissue support as evidenced by clinical attachment loss (CAL) and radiographically determined alveolar bone loss, as well as the presence of periodontal pocketing and gingival bleeding. Periodontitis is a significant public health concern due to its high prevalence, risk of tooth loss and functional impairment, effects on chewing and aesthetics, and its broader impact on social disparities and quality of life.^4^

Corresponding to gingivitis and periodontitis, peri-implant mucositis and peri-implantitis are characterized by inflammatory processes around the implant.^5^^,^^6^ Visual symptoms of inflammation and bleeding on probing are the defining characteristics of peri-implant mucositis. There is substantial evidence that plaque is the primary source of peri-implant mucositis; thus, the elimination of plaque can reverse the peri-implant mucositis. Peri-implantitis is a plaque-associated pathologic condition that manifests as inflammation in the peri-implant mucosa and a consequent progressive loss of supporting bone in the tissue surrounding dental implants. It is presumed that peri-implant mucositis precedes peri-implantitis. Peri-implantitis is linked to people who have a history of severe periodontitis and those who have inadequate plaque control.^7^

The etiology of periodontal and peri-implant diseases depends on bacterial plaque, microbial by-products, and the host immune response.^8^ The bacterial biofilm formation on the tooth surface or implant initiates gingival inflammation, which leads to the activation of several key molecular pathways that ultimately activate host-derived proteinases with the subsequent loss of clinical attachment.^9^ With the progression of clinical attachment loss, the geometry of the periodontal/peri-implant volume changes significantly and develops into a pathological periodontal/peri-implant pocket.^10^^,^^11^

With clinical attachment loss and subsequent bone defects in periodontitis and peri-implantitis, removing bacteria from the distal apical parts of the advancing pocket becomes progressively more difficult. The constrained geometry and inaccessibility of these areas complicate effective cleaning, as traditional mechanical tools such as scalers and curettes are not able to reach these sites. On the other hand, ultrasound and photoacoustic cleaning offer promising alternatives by leveraging mechanisms such as photoacoustic streaming and cavitation. However, initiating the cavitation process in such confined spaces is challenging due to restricted fluid movement and friction against the pocket walls. Previously, we have shown that photoacoustic cleaning with Er:YAG laser could be possible also in distant and hard-to-reach areas due to the effective transmission of cavitation energy through the wedge system that enables secondary cavitation formation and a possibility of non-contact remote cleaning.^12^

Er:YAG laser-induced photoacoustic treatment is routinely used in endodontics for the effective removal of biofilms from constrained geometries of root canal systems composed of semi-solid–solid interfaces.^13^ Much less, however, is known about its effectiveness in soft-solid interfaces found in periodontal/peri-implant-constrained geometries. Here, the highly water-absorptive light of Er:YAG laser is delivered into water-filled periodontal/peri-implant volume via a small-diameter fiber tip (FT). A rapidly dissipating primary cavitation bubble is formed near the FT that triggers pressure disturbances, which in turn leads to secondary cavitation.^12^ Recently, we have shown that biofilms can be effectively removed from the soft-solid wedge boundary mimicking the gingival sulcus.^14^ In this work, we extend the modeling to include the clinical attachment loss of the periodontal/peri-implant pocket. The geometry of the periodontal pocket in clinical conditions is very constrained and essentially forms a zero-gap geometry. It is currently unknown how effective the removal of biofilm from the zero-gap geometry can be. Consequently, the objective of the current study was to assess the effectiveness of biofilm removal from a zero-gap model using Er:YAG laser photoacoustic streaming.

## 2 Materials and Methods

To simulate a periodontal/peri-implant pocket, we manufactured a model system composed of a viscoelastic material in contact with a solid glass and/or titanium surface. In the model, the soft tissue is mimicked by 10% PDMS (polydimethylsiloxan) with a storage modulus of 29,000 Pa,^12^ which is non-covalently attached to the solid glass and/or titanium surface covered with biofilms. Due to the weak adhesion of PDMS, there was no gap between the soft wall and the infected implant and/or titanium surface. We used the laser’s ultra-short pulse modality (USP) to generate a single primary cavitation bubble at different depths within the zero-gap model. The evolution of the primary cavitation bubble at different depths in the zero-gap model system was determined. The effectiveness of biofilm removal from the titanium surface in the zero-gap model system was correlated to the position of the FT and the cavitation bubble evolution.

### 2.1 Zero-Gap Model System

**Fig. 1:**
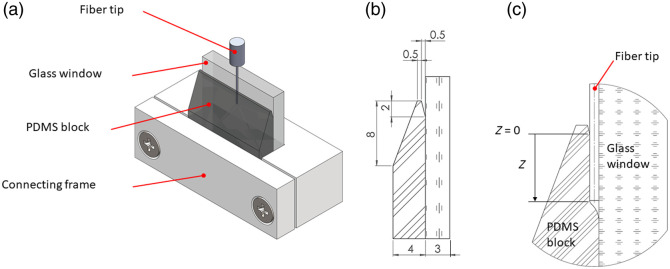
(a) Zero-gap model system. (b) Side view of the PDMS block. All dimensions are in mm. (c) Side view of the zero-gap model system with inserted fiber tip, which creates a small cavity at the end of the fiber tip and at the sides. At Z=0  mm, we have a boundary that separates the upper wedge part (with a negative Z value) and a lower zero-gap part (with a positive Z value).

The model system simulating clinical attachment loss is shown in Fig. 1. Compared with the wedge model system in the previous study,^14^ here, in the zero-gap model system, we have used a significantly smaller wedge and increased the area of PDMS attachment to titanium or glass. This is important if one wants to model clinical attachment loss, where the periodontum or peri-implant gingiva is weakened and partially attached. The model presented is a significantly improved version of the previous model in the following respects: (i) here, we have used a titanium surface instead of a glass surface; (ii) the size of the wedge part is significantly smaller (i.e., wedge depth of 2 mm relative to 7 mm in the previous work) which is more relevant for the clinical case; (iii) width of the PDMS slab was thinner relative to the previous one, which is a better representation of the gum tissue; (iv) most importantly, fiber tip was inserted between the PDMS and titanium in the zero-gap part of the model which has not been tested before. The model consists of four parts: a PDMS block, a glass window, a Plexiglas plate, and a connecting frame. A PDMS block (20×20×4  mm) was tapered to one end mimicking the free gingiva anatomy in its keratinized masticatory surface and the non-keratinized epithelium on its crevicular and junctional surfaces. On the crevicular surface, a small wedge was made 0.5-mm wide and 2-mm deep representing the apical portion of the gingival sulcus. From the apex of the mimicked gingival sulcus, the PDMS was in direct noncovalent contact with the solid surface (glass or titanium disk), replicating the clinical attachment loss. Titanium grade 5 disks (for biofilm removal studies) or glass slides (for high-speed camera experiments) were used for biofilm growth. The titanium disk with mature biofilm and PDMS block was inserted into the seat of the connecting frame and clamped with a 5-mm-thick Plexiglas plate to prevent fluid from leaking from the sides of the pocket during the laser-induced cavitation experiments (the same as in Volk et al.^14^). The reproducibility of generating the primary cavitation bubble of a given size in the assembled setup was estimated to be 5%. The PDMS (Dow Europe GmbH, Germany) was prepared as described previously.^13^ Briefly, 10 g of PDMS was mixed with a curing agent to obtain a 10% (w/w) mixture, degassed at room temperature for 30 min, poured into the fabrication mold, and baked at 70°C for 1 h.

### 2.2 Biofilm Growth and Visualization

Overnight culture (37°C, 200 rpm) of *Pseudomonas aeruginosa* ATCC 27853 in 5-mL Tryptic Soy Broth (TSB) was centrifuged for 5 min at 4000 RCF. The supernatant was removed and the pellet was resuspended in a 1:1 mixture (total volume of 3 mL) of artificial saliva (Fusayama/Meyer Artificial Saliva, Sigma-Aldrich). TSB was supplemented with 2% sucrose. Titanium grade 5 disks (10×8  mm) were sandblasted and autoclaved. Before being used for bacterial adsorption, titanium disks were placed in a 70-mm Petri dish. On each titanium disk, 100  μL of bacterial suspension was placed and incubated in a climate chamber (Memmert) for 90 min at 37°C, 80% relative humidity. After bacterial adsorption, saline suspension was poured into the Petri dish to cover the titanium disks, thus removing the unbounded bacteria. The titanium disks were transferred to a 12-well plate and poured with 2-mL growth media containing TSB (30  mg/mL), sucrose (20  mg/mL),^15^ artificial saliva (2% (v/v),^16^ and ${\mathrm{FeSO}}_{4}$ ($3\times 10^{-4}\;\mathrm{mg}/\mathrm{mL}$).^17^ Biofilms were grown at 37°C, 100 rpm, for at least three days before being used for laser experiments. The growth medium was changed every second day. The titanium disks with confluently grown biofilm were placed in a 50-mL Falcon tube containing 20-mL saline solution and mildly sonicated in a water bath (normal mode, Promed 50, ASonic) for 5 s to remove unattached cells. After mild sonication, the slides were placed in a Petri dish, stained with 5  μL LIVE/DEAD dye (5  μM SYTO9, 20  μM PI), and incubated for 5 min at room temperature. The discs were observed under a confocal laser scanning microscope—CLSM (Zeiss Axio Observer Z1 equipped with confocal unit LSM 800, Jena, Germany). The images were recorded on two fluorescence channels: a 488-nm laser to acquire green fluorescence (SYTO9 stained cells) and a 561-nm laser to acquire red fluorescence (propidium iodide-stained cells). Two positions of the FT were utilized, upper Z=−1  mm and lower Z=2  mm. The upper FT position was located in the wedge part of the model system, whereas the lower FT was positioned inside the zero-gap part of the model system. To assess the effectiveness of biofilm removal, measurements were taken at the two FT positions; in addition, biofilm removal at Z=−1.5  mm, Z=1  mm, and Z=4.5  mm were also determined. Each position on the titanium disk was observed under CLMS by a Plan-Apochromat 100×/1.4 NA objective (Zeiss) with up to 10 slices per z-stack. The typical z-step was 1  μm for the 100× objective with a field of view of 127.78  μm×127.78  μm.

### 2.3 Biofilm Removal

The assembled zero-gap model system with biofilms was immersed 5 mm into the water bath (400-mL glass beaker with a 3D-printed plastic frame to hold the model in place), which ensured that the pocket was constantly filled with water. The Er:YAG laser system (LightWalker^®^, Fotona d.o.o, Ljubljana, Slovenia) delivered ultrashort pulses with a duration of 25  μs (USP) through an articulated arm and handpiece via laser optical fiber. A holder for the articulated arm was used to ensure reproducibility and accuracy of the vertical FT position. The laser fiber tip was parallel to the titanium or glass surface. The laser wavelength of 2940 nm, the H14 handpiece, with a flat fiber tip of a diameter of 400  μm (FlatSWEEPS400/14, Fotona d.o.o.), and a pulse frequency of 15 Hz was used. The laser pulse energy was set to 20 mJ, and the duration of laser treatment was 10 s in both the upper and lower laser tip positions. For each experiment, at least eight titanium disks were processed.

To observe biofilm removal in real time, the experimental setup was the same as described previously.^12^ Briefly, laser-induced biofilm removal from the glass slide was observed with a high-speed camera (Photron, Fastcam SA-Z) equipped with a 1:1 macro lens (Sigma APO Macro, f=180  mm, F2.8) providing an optical resolution of 20  μm. We used a high frame rate of 100,000 fps at a reduced resolution of 360×384  pixels and a shutter speed of 248 ns. The observation area (∼7.5×7.7  mm) was backlit (through the transparent components of the PI model) with a high-brightness white LED light (HB LED) (Thorlabs, MWWHLP2—3000 K, 1713 mW). The transparent Plexiglas, PDMS, and glass windows provided uniform propagation of light, resulting in a bright image background, whereas the FT and cavitation bubbles appeared dark.

High-speed videos of cavitation development and biofilm cleaning were recorded at five different FT depths, from Z=−2  mm to Z=2  mm. Each recording started 10 s after the beginning of the laser irradiation. Forty laser pulses were recorded, which corresponds to 2.66 s at a repetition rate of 15 Hz.

### 2.4 Biofilm Removal Effectiveness

The individual Z-stack images were stacked and analyzed for the bacterial cell coverage (%) of the surface using Fiji software (version 1.54f). First, a Z-project function was used that merged all images from one Z-stack. We used the same procedure as described previously (Volk et al.^14^). Briefly, a triangle method and an ImageJ measuring function were applied.^18^ The images were converted to binary format (black background, white bacterial cells), with an automatically determined threshold value that separates bacterial cells from the background, and surface coverage was then calculated. The difference in surface coverage before and after the laser treatment was used to determine the percentage of biofilm removal from the titanium disks.

To obtain quantitative data on the biofilm architecture, we used BiofilmQ software (Drescher Lab) as described.^19^ Briefly, each fluorescence channel (488 nm green and 561 nm red) was segmented into pseudo-cell cubes with the Otsu segmentation method, 0.9 sensitivity, and a cube size of 20 vox. Then, both channels were merged. For each pseudo-cell cube detected, a single-object parameter Cube Center Coord in BiofilmQ was used that provides a center coordinate (x,y,z). As a global biofilm parameter, we used Biofilm MeanThickness, which is the mean of the thickness of the biofilm at the x-y position of the object. All Z-stack images were converted to TIF images for each slice, and both channels were merged together using Fiji software. For the cell count in each image, we used Cellpose 2.0, a pre-trained neural network model for biological image segmentation.^20^ In Z-stacks, we segmented each slice individually. Cellpose then automatically detects cells and counts them. The segmentation images were manually inspected for possible errors.

### 2.5 Statistical Analysis

The significance between samples was analyzed with IBM SPSS statistics (version 23) using the nonparametric Mann-Whitney U test. P values less than 0.05 were considered statistically significant.

## 3 Results

**Fig. 2:**
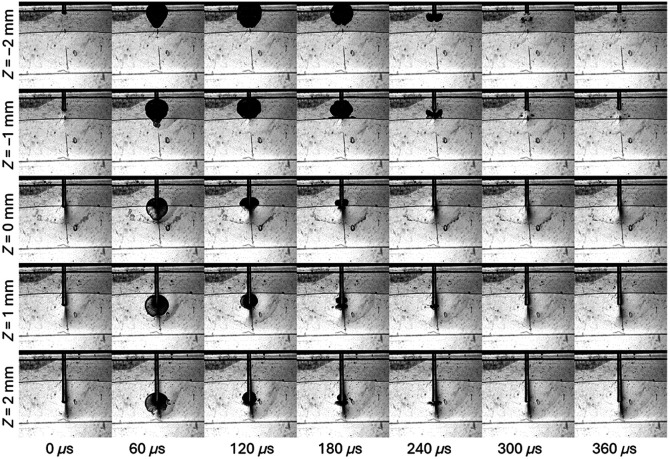
Time evolution of the cavitation bubble as a function of the fiber tip depth (Z). The first two rows (sequences) show cavitation evolution when the FT was located in the wedge part of the pocket. Z=0  mm indicates the FT position at the wedge/zero-gap interface. In the last two rows, the FT was inserted in the zero-gap region of the model system (Video 1, MP4, 4.03 MB [URL: https://doi.org/10.1117/1.JBO.30.2.025002.s1]).

The effect of the laser FT vertical position on the dynamics of the cavitation in the zero-gap model system is shown in Video 1 and Fig. 2. When the FT was positioned at Z=−2  mm (see the first row in Fig. 2), the cavitation bubble developed mainly in the wedge part of the model system and did not cross the wedge/zero-gap interface. When the FT was at Z=−1  mm, a fraction of the cavitation energy was transmitted into the zero-gap part of the model system. When the FT was positioned at the interface between the wedge and the zero-gap part of the model system, most of the cavitation energy was transmitted into the zero-gap part of the model system. Finally, if the FT was positioned inside the zero-gap (Z>0), the cavitation energy was largely contained inside the zero-gap part of the model system, and secondary cavitation bubbles of much smaller dimensions formed in an arc below the primary bubble. These results indicate that the vertical position of the FT has a significant effect on cavitation development, its inhibition, as well as scattering and dispersion of the pressure wave energy in the model system.

**Fig. 3:**
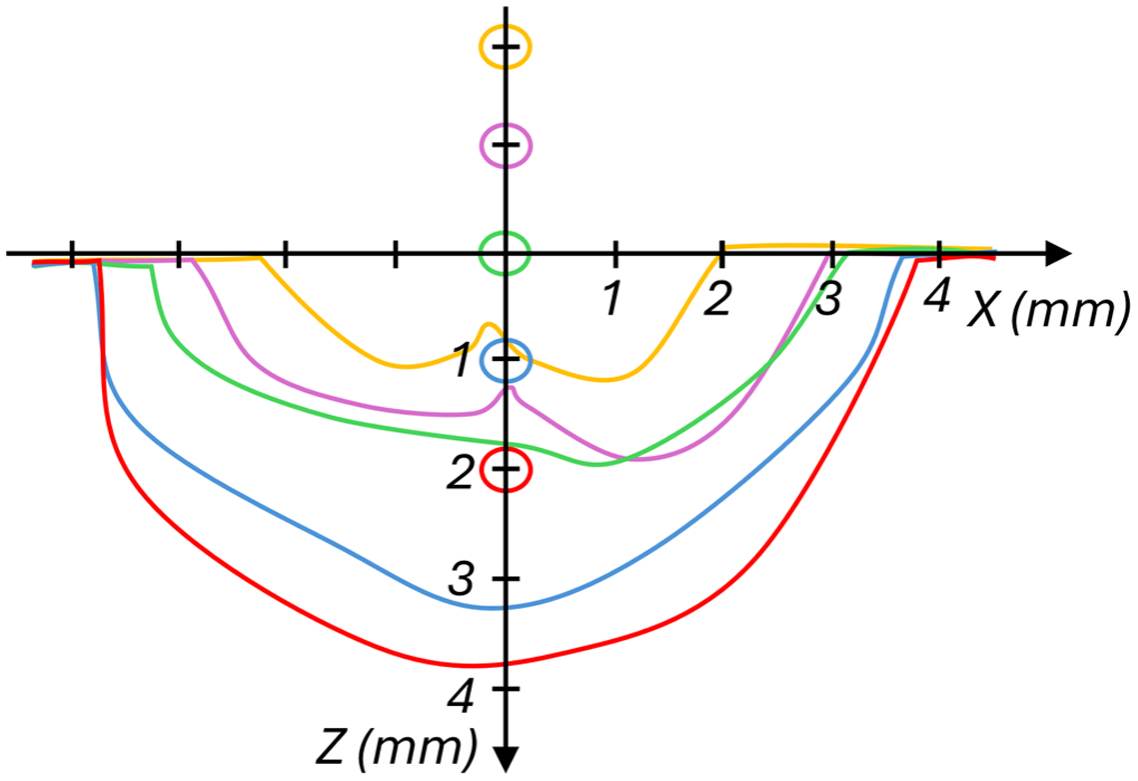
Projection area of the zero-gap opening in the x-z plane (width-height) depending on the position of the FT. The vertical FT position is marked with circles in the same color as the corresponding area border.

A closer inspection of the dynamics in the zero-gap region reveals an opening of the pocket due to the formation of a primary cavitation bubble, which had a characteristic oval shape geometry. Figure 3 shows that the projected area of the opening depends mainly on the vertical FT position. At Z=−2  mm, the opening protrudes ∼1  mm into the zero-gap with a relatively small area affected (Fig. 3). When the FT was positioned inside the zero-gap (Z=2  mm), the width of the projected opened area increased and was ∼7.5  mm wide and 4 mm high.

**Fig. 4:**
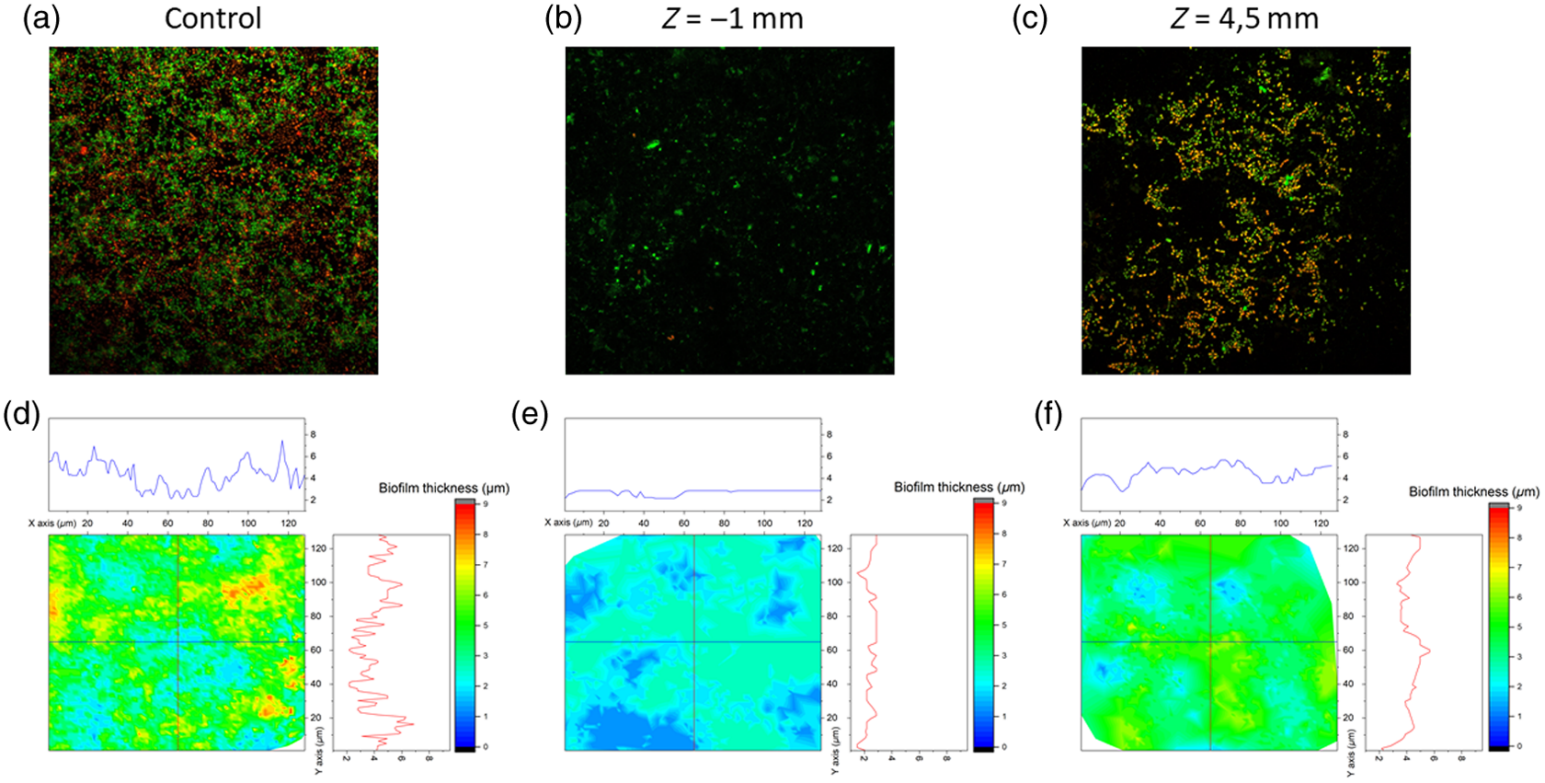
Biofilm removal effectiveness with the FT in the upper position (Z=−1  mm). Representative microscopic images and contour profile graphs of the biofilm prior to the laser treatment (a) and (d), after laser treatment at the position of the FT (Z=−1  mm) (b) and (e), and at the position Z=4.5  mm (c) and (f).

To check how cavitation dynamics in the vertical direction correlate with biofilm cleaning effectiveness, we grew biofilms on the titanium discs and used Er:YAG laser-induced photoacoustic treatment to remove them [Figs. 4(a) and 4(d)]. The biofilms were up to 8.5  μm thick with a rough surface. We observed more than 95% cleaning of the biofilm around the FT in the upper position at Z=−1  mm (the FT located in the wedge part of the model system), in the area that colocalized with the fully expanded cavitation bubble projected onto the biofilm surface. After photoacoustic cleaning, most of the cells in the biofilm around the FT were removed [Figs. 4(b) and 4(e)]. The remaining biofilm had significantly decreased thickness and roughness, with some cell debris and biofilm matrix remnants observed. If the remaining cells were stained with propidium iodide after the laser treatment, the number of dead cells increased. Biofilm cleaning at a distance from location Z=4.5  mm in the zero-gap part of the model was less effective [Figs. 4(c) and 4(f)]. Although the number of attached cells on the surface decreased, most of the titanium surface at Z=4.5  mm was still covered with biofilm. The remaining biofilm was thinner and less rough.

**Fig. 5:**
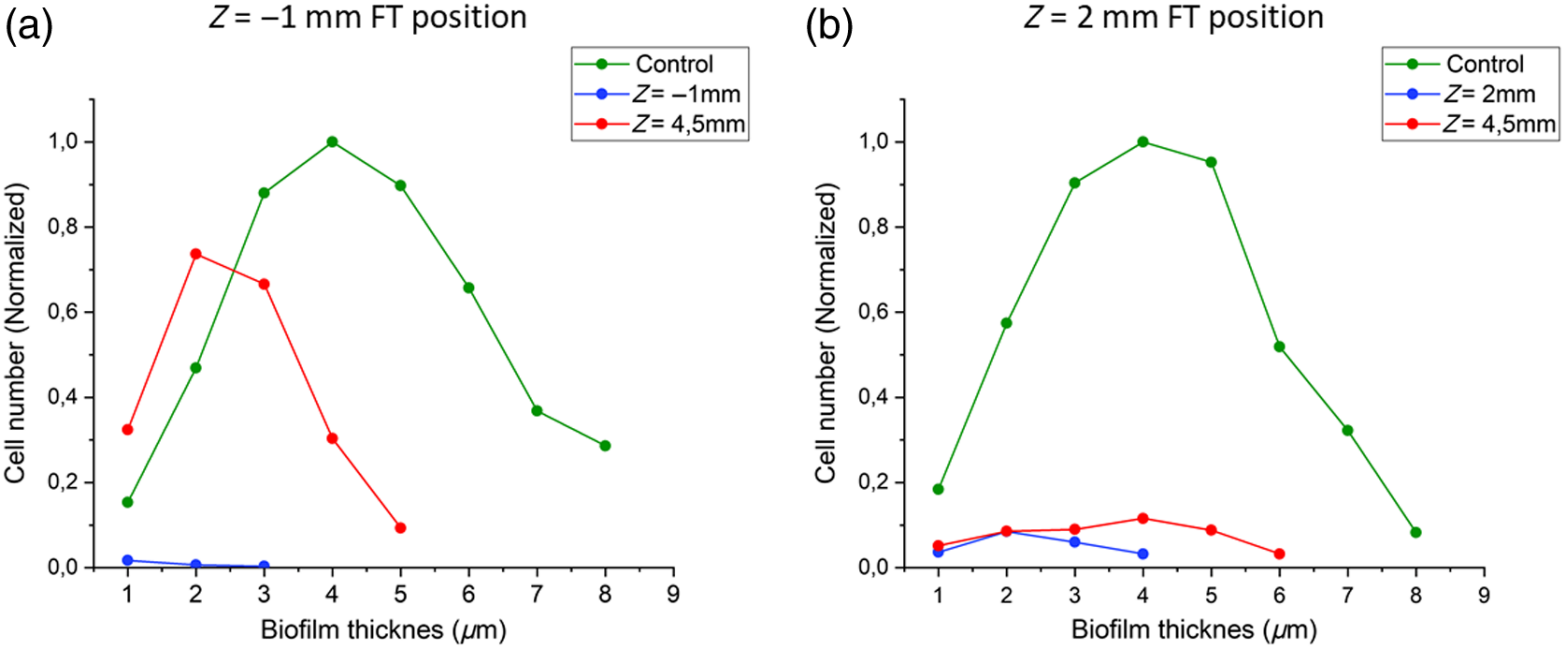
Vertical biofilm cell density represented as a normalized cell distribution in the biofilm in the (a) FT in the upper position Z=−1  mm (inside the wedge part) and (b) FT in the lower position Z=2  mm (inside the zero-gap part) of the model system. Normalized cell density before the laser treatment (green), after laser treatment at the position of the FT (Z=−1  mm, blue), and after laser treatment at location Z=4.5  mm (red). Cell numbers were normalized to the maximum cell density in the control sample.

The cell distribution in the vertical direction in the pretreated biofilm was not uniform [Fig. 5(a)]. The cell density was largest in the center of the biofilm and decreased toward the bottom and the top of the biofilm. After photoacoustic laser treatment, only a few cells were present on the titanium surface near the FT in the wedge part of the model (Z=−1  mm). However, cleaning at a distance inside the zero-gap part was less effective. There, most of the remaining cells were present in a single layer. Some of the remaining cells were present in biofilm patches that were two to four layers thick. On the other hand, when the FT was positioned inside the zero-gap part [Fig. 5(b)], cleaning at the positions of the FT and at a Z=4.5  mm was similarly effective. At both positions, less than 10% of the original cell numbers was observed. More biofilm patches remained at position Z=4.5  mm.

**Fig. 6:**
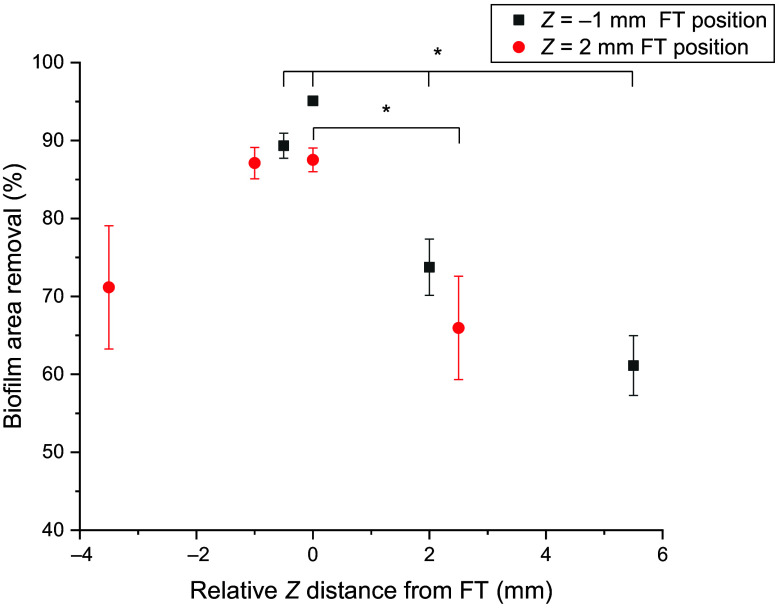
Fraction of the biofilm removed as a function of the distance from the laser fiber tip. The negative distance indicates biofilm cleaning effectiveness above the FT position, position 0 is the position of the FT, and positive distances are locations below the fiber tip. The cleaning effectiveness when the FT was in the upper position (Z=−1  mm) is indicated with black symbols, and the results for the lower FT position (Z=2  mm) are indicated in red. The mean values and standard deviations are given (n=4 for the FT position and n=8 for other positions). The * represents a statistically significant difference between biofilm removal at the FT position and other positions (<p=0.05).

Biofilm cleaning effectiveness depended on the absolute distance from the laser FT (Fig. 6). For both laser positions, it was highest at the position of the FT and decreased significantly above and below the FT. Even at a relatively large distance from the FT (i.e., 5.5 mm from the FT), we were able to remove more than 60% of the biofilm.

## 4 Discussion and Conclusion

Effective removal of biofilm from narrow periodontal and peri-implant pockets is hard to achieve.^21^ In the present study, we used a zero-gap model system composed of soft PDMS material in contact with biofilm grown on a glass or titanium surface to demonstrate the effectiveness of Er:YAG laser-induced photoacoustic removal of biofilm. The results demonstrated that laser treatment can remove bacterial cells very effectively around the laser fiber tip (a log 2.24 reduction in cell number at Z=−1  mm FT and log 1.33 at Z=2  mm FT) in an area equal to the projected surface area of the fully expanded primary cavitation bubble on the biofilm surface. Cleaning was also effective beyond the projected area of the fully expanded primary cavitation bubble; however, the efficacy decreased with the distance from the FT. Although less biofilm was removed when the FT was inside the zero-gap part, more than 85% of the biofilm was still removed when the PDMS and titanium were in contact.

The results suggest a very good correlation between the cleaning of biofilms and the cavitation bubble dynamics. If cavitation is induced in the wedge part of the model system, most of the cavitation energy remains concentrated in the wedge part. The primary cavitation bubble expands radially, but the expansion is hindered toward the wedge/zero-gap interface. It appears that the primary cavitation bubble has only a small penetration ability into the zero-gap of the model system. This corresponds to very good biofilm cleaning in the wedge part but progressively lower cleaning effectiveness in the zero gap. The amount of energy that penetrates the zero gap can be increased by lowering the position of the FT (focusing tube). At lowered FT positions, primary bubble expansion creates space between the PDMS and solid surface. In the opened volume (cavity), the cleaning of the biofilm is effective beyond the projected area of the fully expanded primary cavitation bubble. Opening the zero-gap also allows better cleaning in the wedge part of the model system, as the opened volume directly communicates with the fluid in the wedge part and permits more vigorous mixing in the transiently formed continuous fluid volume.

Similar transient cavity formation can be expected in vivo as well. During bacterial infection, matrix metalloproteinases weaken the attachment of the periodontium or peri-implant gingiva, and in such weakened pockets, laser-induced cavitation could result in transient cavity formation between the soft tissue and cementum or implant. This will allow liquid penetration into the cavity and therefore increased cleaning of the biofilm beyond the size of the fully expanded primary cavitation bubble. Inflamed pockets allow easy penetration of the laser tip, a situation similar to the bleeding on probing test in periodontology. Bleeding on probing is the critical method used in diagnosing periodontal and peri-implant disease. Although there is no consensus on the standard diameter of the rounded probing tip, the probing tip diameters used are between 0.4 and 1.0 mm.^22^ Probe advancement between the gingiva and the tooth/implant is determined by the pressure exerted on the gingival tissues by the dentist, the angle of insertion, the presence of dental calculus, hypersensitivity, and the resistance of the inflamed tissue. The consensus from the literature review is that the pressure used to place the probe tip to the base of the periodontal/peri-implant pocket is $∼50\;N/{\mathrm{cm}}^{2}$ and at the base of the junctional epithelium $200\;N/{\mathrm{cm}}^{2}$.^22^ The diameter of the flat laser fiber tip used in this study was 0.4 mm which is at the lower end of the probes typically used by dentists. By numerical simulation of the deformation of the surrounding soft tissue (see the Supplementary Material), we calculated that the pressure of the cavitation bubble on the soft tissue is $∼5\;N/{\mathrm{cm}}^{2}$. This suggests that the estimated pressure exerted by the cavitation bubble on the soft tissue is at least ten times lower than the pressure applied by the dentist during the probing on a bleeding test. Therefore, we conclude that cavitation may not cause more damage to the soft tissue than the standard bleeding on the probing test. The pressure of the laser pulse on the soft tissue increases with lowering the position of the FT, which allows more energy to concentrate in the zero gap, and consequently, more pressure can be exerted on the soft tissue, which in turn increases the size of the cavity between the soft tissue and cementum or implant surface but consequently also increases biofilm cleaning efficiency.

Another concern during laser treatment is thermal damage. Er:YAG lasers have been well-documented as both effective and safe for periodontal therapy. Even in their directly ablative mode at high energy settings, they minimize thermal effects on surrounding tissues due to their high absorption of water and the simultaneous use of water irrigation. This combination ensures localized and mitigated heat generation, significantly reducing the risk of thermal damage to the surrounding periodontium.^23^ Photoacoustic-induced temperature increases (low-energy settings), as discussed in our manuscript, are substantially lower than those observed in direct laser ablative procedures and are unlikely to reach levels that could compromise tissue integrity.

Recently, the cavitation dynamics and removal of biofilm from a larger wedge model system (height of 7 mm, width of 5 mm, and thickness of 2 mm at the top of the wedge) were studied.^12^^,^^14^ Interestingly, the dynamics of the cavitation bubble evolution did not depend on the FT vertical position, when the position of the FT changed in the upper third of the wedge system.^12^ Consistently, there was only a minor effect on biofilm removal when the position of the FT was changed.^14^ This is very different from the situation in this study, where the wedge was much smaller and similar in size to the apical part of the larger wedge model system. Due to a significantly narrower space, the position of the FT was important both for the dynamics of the primary cavitation bubble evolution as well as for the biofilm cleaning effectiveness. The effect of the FT vertical position was much more pronounced when the FT penetrated into the zero-gap part of the model system and induced geometrical change in the form of the cavity between the PDMS and solid surface.

The results suggest an intimate relation between the geometry of the narrow space, the vertical position of the FT, cavitation bubble evolution, and resulting biofilm removal. When the dimension of the space in which the cavitation develops is much larger than the size of the maximally expanded cavitation bubble (i.e., large wedge model system), the position of the FT does not have a major effect on the primary cavitation bubble dynamics nor the biofilm cleaning effectiveness. When the dimension of the space is similar in size to the maximally expanded primary cavitation bubble (i.e., small wedge model system), the dynamics of the cavitation bubble become distorted, influencing biofilm cleaning effectiveness. On the other hand, when the dimension of the space is much smaller than the unhindered maximally expanded primary cavitation bubble (i.e., zero-gap), the dynamics of the cavitation bubble become severely disturbed. Here, the expanding primary cavitation bubble does work on the surrounding medium to create the cavity. Because in the soft-solid model, only soft tissue can yield cavitation energy, the produced cavity is not symmetric and bulges toward the soft tissue.

In the large wedge model, cleaning of the biofilm at the apex of the wedge system was significantly improved by secondary cavitation bubbles.^12^ No secondary cavitation was detected in the wedge part of the model system used in this study; however, when the FT was immersed in the zero-gap, secondary cavitation was observed below the primary cavitation bubble. As secondary cavitation produces smaller cavitation bubbles, which can penetrate deeper into the zero-gap, this observation can be important and could have significant implications for cleaning the biofilm in difficult-to-reach places. This certainly needs further attention and experimentation.

In dentistry typically threaded implants are used which results in undercut areas under the threads of the implants which presents unique opportunities for photoacoustic cleaning. In direct ablative cleaning, the laser tip must be in direct contact with the target tissue, which limits its effectiveness in reaching areas underneath the threads due to restricted accessibility. By contrast, photoacoustic cleaning effectively overcomes these limitations by harnessing the dynamics of both primary and secondary cavitation, as well as the intricate behaviors of fluid flow and pressure gradients. This innovative approach enables the cleaning of areas that are distant from the tip’s direct insertion point, ensuring more comprehensive biofilm removal. This phenomenon was demonstrated in irrigant flow dynamics in simulated lateral canals, showcasing how high-speed fluid movement can occur in regions not in direct proximity to the inserted tip.^24^ Similar to laser cavitation, Vyas et al. demonstrated the cleaning ability of ultrasound (US) cavitation in effectively removing biofilm from in between implant threads, highlighting the potential of caitation as a non-invasive approach for cleaning hard-to-reach areas in periodontal and peri-implant pockets.^25^

In conclusion, the results suggest that primary cavitation bubble evolution can be significantly influenced by the geometry and softness of the surrounding boundaries, which in turn determine biofilm cleaning effectiveness. In the wedge part of the model system, more than 95% of the biofilm surface and in the zero-gap more than 85% of the biofilm surface were removed at the projected surface area of the fully expanded primary cavitation bubble near the laser’s fiber tip. Promising cleaning effectiveness in the zero-gap extends beyond the projected area of the fully expanded primary cavitation bubble, mainly due to the cavity produced between the soft tissue and solid surface upon expansion of the primary cavitation bubble. It is important to note that the cleaning effect of Er:YAG-induced photoacoustic irrigation extends beyond the fiber-tip position, although it becomes less effective with increasing distance from the fiber tip. This remote cleaning capability is particularly valuable for reaching difficult areas within periodontal and peri-implant pockets and should be examined further.

## Supplementary Material

10.1117/1.JBO.30.2.025002.s01

10.1117/1.JBO.30.2.025002.s1

## Data Availability

All data in the paper will be made available upon reasonable request to the primary authors.
