# Supplementary material for: Er:YAG laser biofilm removal from zero-gap periodontal/peri-implant model system mimicking clinical attachment loss
Source: J Biomed Opt. 2025 Feb 25;30(2):025002. doi: 10.1117/1.JBO.30.2.025002 (PMC11853840; doi:10.1117/1.JBO.30.2.025002)
Supplement: Supplementary file 1 [file JBO_030_025002_SD001.pdf]

Supporting information

1. Numerical simulation of soft-tissue deformation due to expanding cavitation bubble

A three-dimensional static structural simulation was carried out using the finite element method (Ansys 2022 R1 program). The simulated geometry of the zero-gap model was identical to that of the experimental setup (Fig. 1). The position of the FT was at Z = 2 mm, so that the cavitation bubble developed entirely in the area of the zero gap. The diameter of the bubble was 2 mm, which corresponds to the measured diameter of the bubble (Fig. 3). The modulus of elasticity of the PDMS was 2 MPa to simulate a large Young modulus case and the Poisson's ratio was 0.49.[23,24]

Figure S1 shows the simulated geometry with the associated boundary conditions and the compressive load. On the PDMS surface, which forms a zero gap with the adjacent solid wall, we considered just the compressive load. Only one half of the model was modeled due to symmetrical geometry. The size of the final elements (tetrahedron) was 0.5 mm.

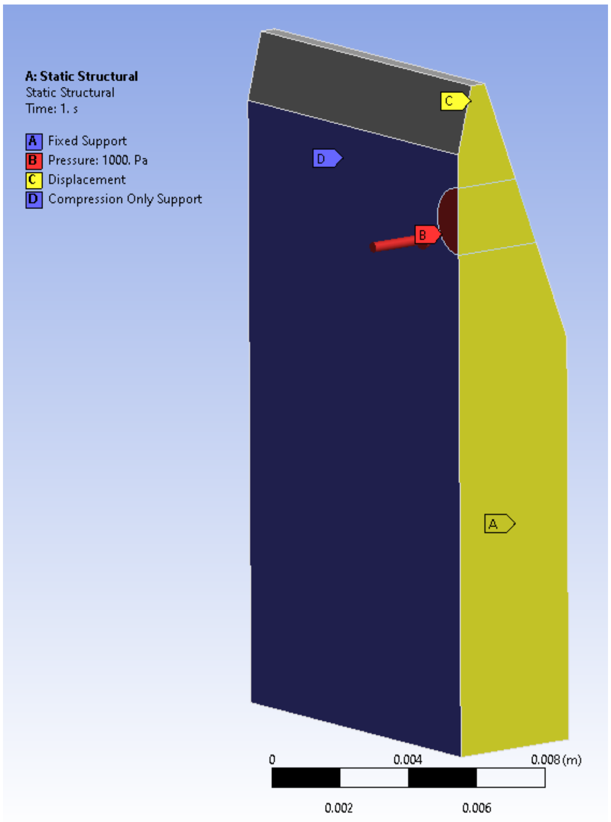

Figure S1. Simulated geometry of a zero-gap model with boundary conditions. Due to symmetry along the vertical plane (arrow A) only one half of the model was modelled. Front

and back supporting plates are replaced with fixed supports (arrows C and D). A pressure load was applied to the circular area (arrow B).

The pressure was increased until the cavity of the same size as the experimentally measured cavity (width 7.5 mm) was achieved. We measured the spreading width only when this was greater than 0.05 mm. Figure S2 shows normal deformation of the PDMS's front surface at a pressure of 5 N/cm^2, at which the spread width was 7.5 mm.

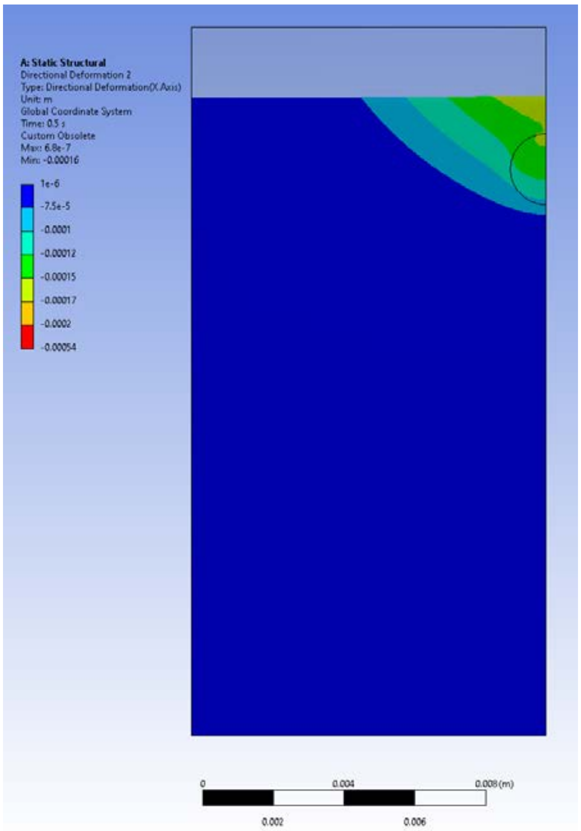

Figure S2. Normal deformation of the PDMS's front surface at a pressure of 5 N/cm^2.
